# Optimizing HIV case identification: investigating client characteristics predictive of HIV positivity from provider-initiated testing (PITC) in central Kenya

**DOI:** 10.1186/s12913-023-09876-9

**Published:** 2023-09-19

**Authors:** Rachael Muinde, Kevin Owuor, Jones Mutiso, Jonathan Mwangi, Paul Wekesa

**Affiliations:** 1 Centre for Health Solutions, Nairobi, Kenya; 2 Division of Global HIV & TB, Center for Global Health, US Centers for Disease Control and Prevention, Nairobi, Kenya

**Keywords:** HIV testing, Positivity, Provider Initiated Testing, Client characteristics

## Abstract

**Background:**

Routine program data indicates positivity rates under 2% from HIV testing services (HTS) at sites supported by Centre for Health Solutions-Kenya in Central Kenya. Achieving the UNAIDS 95:95:95 goals requires continuous identification of people living with HIV in an environment of diminishing resources. We assessed non-clinical and clinical characteristics of persons who tested HIV-positive aimed at improving the process of HTS through Provider-Initiated HIV Testing & Counseling (PITC).

**Methods:**

We conducted a retrospective analysis of routine PITC program data collected between October 2018 and September 2019 from six health facilities located in three counties in central Kenya. Stratification was based on county and facility volume. A multivariable logistic regression model, clustered adjusted for facility using robust standard errors, was used to determine predictors of a positive HIV result.

**Results:**

The total sample was 80,693 with an overall positivity rate of 1.2%. Most, (65.5%), were female and 6.1% were < 15 years. Most clients, 55,464 (68.7%), had previously tested for HIV. Client characteristics associated with a higher odds of positivity on multivariable analysis included: being female (adjusted odds ratio [aOR] 1.27, 95% confidence interval [CI] (1.03–1.57); adults 15 years and above compared to children < 15 years, divorced and married polygamous compared to married monogamous [aOR 3.98, 95% CI (2.12–7.29) and aOR 2.41 95% CI (1.48–3.94) respectively]; clients testing for the first time compared to repeat testers in less than 12 months [aOR 1.39, 95% CI (1.27–1.51)]. Similarly, repeat testers in more than 12 months compared to repeat testers in less than 12 months [aOR 1.90, 95% CI (1.55–2.32)]; presumptive TB clients compared to those without signs of TB [aOR 16.25, 95% CI (10.63–24.84)]. Clients tested at inpatient departments (IPD) were more likely to get a positive HIV result compared to those tested at outpatient departments (OPD), and other departments.

**Conclusions:**

The study findings highlight client characteristics such as age, marital status, HIV test entry point, first-time test, repeat test after 12 months, and TB status as factors that could influence PITC results and could be used to develop a screening tool to target eligible clients for HTS in low HIV prevalence settings.

## Introduction

An estimated 1.5 million Kenyans are living with HIV of whom 79.5% are aware of their status [1]. The 2018 Kenya HIV prevalence survey (KENPHIA), estimated a national HIV prevalence of 4.9% among adults and 0.7% among children. In three counties of central Kenya, HIV prevalence was estimated at 4.2% in Murang’a County, 2.2% in Nyandarua, and 5.1% in Nyeri County [1]. Compared to the high HIV burden regions such as Homa Bay County with a prevalence rate of 19.6%, these are low HIV-burden counties and also categorized as middle antiretroviral treatment (ART) coverage counties ranging between 70 to 79% [2]. Routine program data indicates continued low positivity rates from HIV testing services (HTS) with the three counties reporting positivity rates below 2% (male 1.8% and female 1.5%). In 2021, the highest positivity was reported among adults above 25 years (2%) and children aged 0 to 4 years (1.4%) [3].

Achieving the UNAIDS first and second 95 goals requires identifying people living with HIV (PLHIV) and linking them to treatment [4]. Getting to the 95% target remains an uphill task as factors associated with HIV infection continue to change and therefore the need to continuously improve our understanding of the HIV epidemic by region and population. These constant changes suggest the need to continually review program implementation strategies for HTS approaches toward meeting the UNAIDS goals [5]. To this end, the United States President’s Emergency Fund for AIDS Relief (PEPFAR) Country Operation Plan 21 required implementing partners to optimize the limited resources by adopting efficient interventions for HIV case identification. Self-test, social network strategies (SNS), and assisted partner notification services (aPNS) are recommended as efficient HTS approaches in Kenya [2, 6]. The 2020 PEPFAR report recognized the need to advance provider-initiated testing and counseling (PITC) as a strategy that contributes the highest number of HIV positives identified compared to other HTS modalities and remains one of the least costly case-finding strategies available [7]. The WHO HIV testing guidelines of 2019 also recommend targeted HIV testing through the use of a symptom screening approach in low HIV burden settings with a national HIV prevalence of below 5% [8].

Research evidence suggests that the implementation of risk prediction algorithms based on patient characteristics could be used to strengthen risk screening and improve case identification and thus introduce moderate efficiencies to HIV testing services offered in health facilities [9]. The 2016 Kenya HIV testing services guidelines which recommended a routine opt-out PITC approach also provided generalized categories of high-risk persons to be prioritized for a test. The national program further proposed the integration of HTS in all service delivery points for national implementation [10]. However, to achieve efficiency in PITC there is a need for deeper analysis to further understand program outcomes based on non-routine parameters and their impact in a sub-national context. Since 2017, the Centre for Health Solutions – Kenya (CHS) has implemented aPNS in central Kenya alongside other effective strategies that continue to yield good results measured by the number needed to test to get one HIV-positive case. The program data however shows that PITC contributes over 60% of cases identified in the region. Our study aimed to describe the demographic and clinical characteristics of persons who tested HIV positive to improve the efficiency of HTS to test less and identify more through PITC in Murang’a, Nyandarua, and Nyeri counties of central Kenya.

## Study methods

### Study population

The study population was all persons tested for HIV through routine provider-initiated testing and counselling interventions. Persons who tested through community testing or partner notification services were excluded from the analysis.

### Study design and setting

This was a cross-sectional retrospective study utilizing routine HTS data from October 2018 to September 2019. Six facilities located across 3 counties in central Kenya including Murang’a (Population 1,056,640; HIV prevalence 3%, ART coverage 91%), Nyandarua (Population 638,289; HIV prevalence 2.2%, ART coverage 90%), and Nyeri counties (Population 759,164; HIV prevalence 5.1%, ART coverage 91%) were included in the study [1, 11]. These are Ministry of Health (MOH) owned county referral and primary healthcare hospitals that are supported by the Centre for Health Solutions – Kenya (CHS) to provide HTS services through the Tegemeza Plus project, with funding and technical assistance from the US Centers for Disease Control and Prevention (CDC). Other details, including outcomes of this project setting, have been described elsewhere [12–16]). The facilities were purposively sampled to include a mix of high volume (facility 1—County, facility 2—County, facility 3—County) and low volume (facility 1—County, facility 2- County, facility 3—County). Facility categorization was based on the monthly outpatient department (OPD) workload with high volume being > 10,000 patients seen in a month and low volume being < 10,000 patients seen in a month. A census of all clients was done and included a total of 80,683 clients (35,018 – Murang’a county, 21,909 – Nyandarua county, and 23,756 – Nyeri county).

### Data collection and management

Routine data were collected by data officers who abstracted data from the laboratory HIV testing services registers (MOH 362) covering the period between October 2018 to September 2019. The outcome variable was the HIV test result while predictors variables included the date of the HIV test, age, sex, marital status, population type (key population [men who have sex with men (MSM), people who inject drugs (PWID), female sex workers (FSW)] versus general population), department (outpatient department [OPD] vs inpatient department [IPD], integrated management of childhood illnesses (IMCI), others [Medical outpatient clinics for dermatology, dental, diabetes]), new (never tested before) or repeat HIV test (tested in the last 12 months), duration since last HIV test, history of HIV test, history of sexually transmitted infections (STI), tuberculosis (TB) screening results, pre-exposure prophylaxis (PrEP) screening results (answering ‘Yes’ to any question in the PrEP RAST tool) and GBV screening result (answering ‘Yes’ to any question in the National GBV screening criteria). Data were entered into a MySQL database and cleaned by data officers, and the final dataset was exported in a Microsoft Excel format for analysis.

### Statistical analysis

Routine HIV individual testing data collected at health facilities were used. Descriptive statistics included mean (standard deviation) and counts (proportions). Univariable and multivariable logistic regression models, clustered adjusted for facility-level variations, were used to determine predictors of a positive HIV result. The final multivariable model used was developed using a backward stepwise approach with the probability of inclusion set at 0.20. The univariable and multivariable odds ratios (aOR) and corresponding 95% confidence intervals (CIs) were presented. All the statistical tests were evaluated at the 5% level of significance. All the analyses were done in Stata version 15.1 (StataCorp. 2017. Stata Statistical Software: Release 15. College Station, TX: StataCorp LLC.).

### Ethical approval

This study was approved by the Kenyatta National Hospital Ethics and Scientific Research Committee. The protocol was also reviewed in accordance with the U.S. Center for Disease Control and Prevention (CDC) human research protection procedures and was determined to be research, but the CDC investigators did not interact with human subjects or have access to identifiable data or specimens for research purposes. We received a waiver of informed consent for the use of retrospective data. All data were kept confidential and only the CHS team had access to identifiable patient data.

## Results

### Socio-demographic characteristics of clients

A total of 80,683 clients had an HIV test done between October 2018 to September 2019 as shown in Table 1. Of these, 52,878 (65.5%) were female, 4,894 (6.1%) were less than 15 years old, and 50,016 (62.0%) were in a married monogamous relationship. More than two-thirds 55,464 (68.7%) had previously tested for HIV.

**Table 1:** Description by HIV positive status among clients in central Kenya, October 2018 to September 2019

	**HIV test result**		
**Characteristics**	**Negative**	**Positive**	**Total**
n (%)	79,701 (98.8)	982 (1.2)	80,683 (100.0)
County			
Murang’a	34,600 (43.4)	418 (42.6)	35,018 (43.4)
Nyandarua	21,565 (27.1)	344 (35.0)	21,909 (27.2)
Nyeri,	23,536 (29.5)	220 (22.4)	23,756 (29.4)
Age (Years), mean (sd)	35.2 (15.4)	37.3 (13.5)	35.2 (15.3)
Age category (Years)			
< 15	4861 (6.1)	33 (3.4)	4894 (6.1)
15–19	5152 (6.5)	31 (3.2)	5183 (6.4)
20–24	11,069 (13.9)	91 (9.3)	11,160 (13.8)
25–29	11,061 (13.9)	123 (12.5)	11,184 (13.9)
30–34	10,651 (13.4)	163 (16.6)	10,814 (13.4)
35–39	8966 (11.2)	158 (16.1)	9124 (11.3)
40–44	7297 (9.2)	111 (11.3)	7408 (9.2)
45–49	5309 (6.7)	80 (8.1)	5389 (6.7)
50 +	15,335 (19.2)	192 (19.6)	15,527 (19.2)
Marital Status, n (%)			
Married Monogamous	49,456 (62.1)	560 (57.0)	50,016 (62.0)
Married Polygamous	228 (0.3)	6 (0.6)	234 (0.3)
Divorced	1942 (2.4)	93 (9.5)	2035 (2.5)
Separated	918 (1.2)	11 (1.1)	929 (1.2)
Single	27,157 (34.1)	312 (31.8)	27,469 (34.0)
Sex, n (%)			
Male	27,487 (34.5)	318 (32.4)	27,805 (34.5)
Female	52,214 (65.5)	664 (67.6)	52,878 (65.5)
Population Type, n (%)			
General Population	79,645 (99.9)	981 (99.9)	80,626 (99.9)
Key Population	56 (0.1)	1 (0.1)	57 (0.1)
Department Testing, n (%)			
IMCI	2867 (3.6)	14 (1.4)	2881 (3.6)
IPD	4359 (5.5)	149 (15.2)	4508 (5.6)
OPD	71,692 (90.0)	813 (82.8)	72,505 (89.9)
Other	783 (1.0)	6 (0.6)	789 (1.0)
Test type, n (%)			
Repeat Test in < = 12 months	46,465 (58.3)	482 (49.1)	46,947 (58.2)
Repeat Test in > 12 months	8359 (10.5)	158 (16.1)	8517 (10.6)
Never Tested	24,877 (31.2)	342 (34.8)	25,219 (31.3)
GBV, n (%)			
GBV- None detected	79,436 (99.7)	979 (99.7)	80,415 (99.7)
GBV- Physical/Sexual violence	265 (0.3)	3 (0.3)	268 (0.3)
TB, n (%)			
No signs	79,167 (99.3)	909 (92.6)	80,076 (99.2)
Not done	15 (0.0)	0 (0.0)	15 (0.0)
TB Treatment	159 (0.2)	6 (0.6)	165 (0.2)
Presumed TB	360 (0.5)	67 (6.8)	427 (0.5)
PrEP Eligibility, n (%)			
Not Eligible	79,512 (99.8)	977 (99.5)	80,489 (99.8)
Eligible	189 (0.2)	5 (0.5)	194 (0.2)

*Sd* standard deviation

### HIV testing outcome

The overall HIV positivity rate among the clients tested was 1.2% (982/80,683). HIV positivity rate was 1.4% (*n =* 342) among the 25,219 first-time testers, 1.0% (*n =* 482) among those who had previously tested (‘repeat testers’) for HIV within 12 months, and 1.9% (*n =* 158) among the repeat testers in over 12 months. The highest positivity rates were among ages 25–34 [286 (1.3%)], 35–44 [269 (1.6%)], and 45–54 [164 (1.6%)] as shown in Table 1.

### Client characteristics associated with a positive test

#### Univariable analysis

On univariable analysis, adults aged 25 to 29, 30 to 34, 35 to 39, and 40 to 49 years compared to children less than 15 years were associated with significantly higher odds of having a positive HIV test result (Table 2). Divorced and married polygamous clients compared to married monogamous ones had higher odds of having a positive HIV result, OR 2.41, 95% CI (1.48–3.94) and OR 3.98, 95% (2.12–7.29) respectively. Female clients compared to males also had higher odds of having a positive HIV test result, OR 1.27, 95% CI (1.03–1.57). Clients tested at IMCI, outpatient, or other departments compared to IPD were less likely to have a positive HIV test result [OR 0.14 95% CI (0.12–0.17), OR 0.33 95% CI (0.20–0.55), and OR 0.22 (0.19–0.26) respectively] (Table 2).

**Table 2:** Factors associated with a positive HIV test result among clients in central Kenya, October 2018 to September 2019

Outcome: Positive vs. Negative HIV test result	Univariable Odds Ratio (95% CI)	*P* value	Multivariable Odds Ratio (95% CI)^a^	*P* value
County				
Murang’a	Reference		Reference	
Nyandarua	1.32 (0.62–2.82)	0.473	1.28 (0.70–2.34)	0.418
Nyeri	0.77 (0.36–1.65)	0.508	0.73 (0.42–1.28)	0.273
Age category (Years)				
< 15	Reference		Reference	
15–19	0.89 (0.51–1.55)	0.672	0.99 (0.56–1.75)	0.961
20–24	1.21 (0.77–1.90)	0.406	1.65 (1.01–2.69)	0.044
25–29	1.64 (1.18–2.27)	0.003	2.45 (1.61–3.74)	< 0.001
30–34	2.25 (1.24–4.10)	0.008	3.40 (1.54–7.51)	0.003
35–39	2.60 (1.49–4.52)	0.001	3.75 (1.79–7.83)	< 0.001
40–44	2.24 (1.24–4.06)	0.008	3.16 (1.56–6.43)	0.001
45–49	2.22 (1.29–3.83)	0.004	3.06 (1.64–5.70)	< 0.001
50 +	1.84 (1.04–3.27)	0.036	2.57 (1.27–5.17)	0.009
Marital Status				
Married Monogamous	Reference		Reference	
Married Polygamous	2.32 (1.53–3.53)	< 0.001	2.41 (1.48–3.94)	< 0.001
Divorced	4.23 (2.28–7.85)	< 0.001	3.98 (2.17–7.29)	< 0.001
Separated	1.06 (0.63–1.78)	0.831	0.96 (0.54–1.73)	0.900
Single	1.02 (0.74–1.39)	0.928	1.50 (0.92–2.47)	0.106
Sex				
Male	Reference		Reference	
Female	1.10 (0.86–1.41)	0.452	1.27 (1.03–1.57)	0.028
Population Type				
General Population	Reference			
Key Population	1.45 (0.07–31.19)	0.812		
Department Testing				
IMCI	0.14 (0.12–0.17)	< 0.001	0.15 (0.10–0.24)	< 0.001
IPD	Reference		Reference	
OPD	0.33 (0.20–0.55)	< 0.001	0.26 (0.13–0.54)	< 0.001
Other	0.22 (0.19–0.26)	< 0.001	0.26 (0.14–0.46)	< 0.001
Test type				
Repeat Test in < = 12 months	Reference		Reference	
Repeat Test in > 12 months	1.82 (1.32–2.52)	< 0.001	1.90 (1.55–2.32)	< 0.001
Never Tested	1.33 (1.09–1.62)	0.006	1.39 (1.27–1.51)	< 0.001
Gender-Based Violence				
GBV- None detected	Reference			
GBV-Physical/Sexual violence	0.92 (0.13–6.67)	0.933		
Tuberculosis				
No signs	Reference		Reference	
Not done	-	-	-	-
TB Treatment	3.29 (0.55–19.69)	0.193	2.77 (0.65–11.87)	0.169
Presumed TB	16.21 (8.32–31.57)	< 0.001	16.25 (10.63–24.84)	< 0.001
PrEP Eligibility				
Not Eligible	Reference		Reference	
Eligible	2.15 (0.84–5.51)	0.110	1.77 (0.82–3.80)	0.147

^a^Backward stepwise model selection used

Newly tested clients and those having a repeat test after 12 months compared to < 12 months had significantly higher odds of having a positive HIV test result (OR 1.33, 95% CI (1.09–1.62) and (OR 1.82, 95% CI (1.32–2.52) respectively as shown in Table 2. The presumptive TB clients compared to clients with no signs of TB had significantly higher odds of having a positive HIV test result, (OR 16.21, 95% CI (8.32–31.57) (Table 2).

#### Multivariable analysis

On multivariable analysis, being an adult (15 years plus) aged, 20 to 24, 25 to 29, 30 to 34, 35 to 39, 40 to 49, and 50 plus years compared to children less than 15 years was associated with significantly higher odds of having a positive HIV test result, [aOR 1.65 95% CI (1.01–2.69), aOR 2.45 95% CI (1.61–3.74), aOR 3.40 95% CI (1.54–7.51), aOR 3.75 95% CI (1.79–7.83), aOR 3.16 95% CI (1.56–6.43), aOR 3.06 95% CI (1.64–5.70), and aOR 2.57 (1.27–5.17) respectively] as shown in Table 2. Female clients had significantly higher odds of having a positive HIV test result compared to males (aOR 1.27, 95% CI (1.03–1.57). Divorced clients and married polygamous compared to married monogamous ones also had significantly higher odds of having a positive HIV result [aOR 3.98, 95% CI (2.12–7.29) and aOR 2.41 95% CI (1.48–3.94)] respectively. Those clients tested in IMCI, OPD, or other departments were still less likely to have a positive HIV test result using IPD as a reference, [aOR 0.15 95% CI (0.10–0.24), aOR 0.26 95% CI (0.13–0.54), and aOR 0.26 (0.14–0.46)] respectively as shown in Table 2.

New clients testing for the first time compared to repeat testers in less than 12 months had significantly higher odds of having a positive HIV test result (aOR 1.39, 95% CI (1.27–1.51). Similarly, repeat testers in more than 12 months compared to repeat testers in less than 12 months also had significantly higher odds of having a positive HIV test result (aOR 1.90, 95% CI (1.55–2.32). Presumptive TB clients compared to those with no signs of TB had significantly higher odds of having a positive HIV test result (aOR 16.25, 95% CI (10.63–24.84)), as shown in Table 2.

Key populations (MSM, PWID, FSW) and GBV status did not meet the model probability inclusion criteria and were therefore not included in the final multivariable model.

## Discussion

Our study looked at the demographic and clinical characteristics of persons who tested HIV positive in central Kenya. Older clients aged over 20 years and above had significantly higher odds of testing HIV positive compared to those below 15 years. Among all age groups, this study showed that clients aged 35–39 years had the highest odds of testing HIV positive as compared to clients below 15 years. This finding differs from a study carried out in rural Kenya and Uganda which showed that more HIV infections were among persons aged 15–34 years [1, 13] and the KENPHIA 2018 report which showed that prevalence peaked among adults aged 45 to 49 years. The reason for this difference may be explained by the geographical differences in the HIV epidemic between Kenya and Uganda, with Uganda being classified under countries with > 10% adult prevalence as compared to Kenya which is classified under countries with adult prevalence of between 1–5% [5]. Another reason for the differences could be that the KENPHIA assessment covered the entire country whereas this study was only conducted in three counties in central Kenya.

Our study showed that the divorced and married polygamous were more likely to test HIV-positive compared to the married monogamous. This is consistent with a systematic review done in seven sub-Saharan African countries and three other studies carried out in Kenya [11, 17–21] which reported that the divorced/separated were significantly more likely to be HIV-positive compared to the married. While marital status may not be a prominent indicator in HIV eligibility screening tools, our study suggests its potentially important role in improving yield and should be incorporated in risk assessment for testing in Kenya and similar settings.

Female clients seeking health services were more likely to test HIV-positive as compared to male clients. This corroborates a finding of a study carried out in rural Kenya and Uganda and population-based HIV impact assessments done in Kenya, Uganda, and Tanzania which reported that women comprised of a majority of those likely to test HIV-positive [17]. This further corroborates population-based HIV impact assessments (PHIAs) done in Kenya, Uganda, Tanzania, and Rwanda that indicated that more females were HIV infected than males [1, 21–23]. This could be because of better health-seeking behavior among females than males, coupled with female biological and socio-cultural vulnerabilities, such as a lack of power to bargain for condom use during sex [24]. For this reason, service delivery points visited by female clients could be strengthened by including an HTS provider offering testing services to maximize testing and yield.

Patients tested at IPD were more likely to test HIV positive as compared to those tested at IMCI and other departments. This is consistent with a finding of a study carried out in several sub-Saharan countries which reported that PITC among inpatients had the highest positivity rate compared to other testing departments [25]. This is because clients already admitted in hospitals (if HIV infected) would likely show symptoms of HIV infection and be easy to reach hence the explanation for the high positivity rate compared to other departments. This means that for effective yield in inpatient departments, strategies could be put in place to screen all admitted patients for HTS eligibility and offer ‘opt-out’ HIV testing to all those eligible while addressing concerns about privacy and stigma in crowded spaces [25]. Patient education on the need to test for those admitted for other illnesses could be encouraged to reduce the chances of opting out of the HIV test.

Clients testing for the first time were more likely to test HIV-positive as compared to repeat testers. This reflects similar findings from two studies done in Kenya which showed that HIV-positive results were more common among first-time testers [18, 26]. This is explained by some factors such as the client’s location far from the testing facility, lower age bracket of 18–24, and low education level [27]. Including questions in HIV screening tools that explore why first-time testers do not test when they visit a health facility could help to improve access.

Clients having a repeat test after 12 months were more likely to test HIV-positive compared to those having a repeat test in less than 12 months. This concurs with two studies done in Kenya which showed that HIV-positive test results were most common among first-time testers and late re-testers [24]- 27. With frequent testing, clients receive prevention messages during the testing and counseling session and are likely to follow the prevention measures given. This could be a possible reason why repeat testers after 12 months are likely to test HIV-positive as compared to those who repeat tests in less than 12 months. Programs in Kenya and similar settings should optimize HTS eligibility screening to identify first-time testers and late retesters to be prioritised for HIV testing to improve yield. Stakeholders could also educate clients to increase awareness of the need for HIV testing for those never tested and at risk and among late retesters.

Presumptive TB clients were more likely to test HIV-positive compared to those with no signs of TB. This finding is consistent with studies done in Nairobi, Kenya, and India which showed that persons with symptoms of TB had an HIV prevalence of 61% [28] and a 12% yield respectively among patients with presumptive TB [29]. This is because TB is among the major opportunistic infections in HIV-infected persons [18, 19]. This means that all clients presenting at the health facilities with signs suggestive of TB need to be screened for HTS eligibility with fidelity and tested. This also calls for streamlining patient flows in TB clinics to ensure all patients in such departments are tested for HIV, reducing missed opportunities. Programs in Kenya and similar settings should optimize presumptive TB screening as an important opportunity to increase yield from HIV testing.

GBV status was not significantly associated with HIV positivity in this study. This however contrasts with a study carried out in the South Wollo zone, Ethiopia which reported that partner sexual violence by another perpetrator was strongly associated with HIV infection [30]. There is a need to tailor-make the eligibility screening process for individuals undergoing GBV, who are at ongoing risk for HIV infection in the central region of Kenya.

PrEP eligibility was not significantly associated with HIV positivity in this study. This is central to the WHO recommendation that PrEP be offered to populations at substantial ongoing risk. This calls for further study on PrEP eligibility and HIV positivity in different settings.

Key populations (MSM, FSW, PWID) did not show any significant association with HIV positivity. The limitation in measurement of this particular variable is that this study was carried out in a general population setting with only 57 clients identified as key population, and only one turning out to be HIV-positive; hence, could give a misleading picture because of the low numbers used. This differs from a report by UNAIDS that reported the rate of new adult infections among the key population and their sexual partners was 62%. Key populations are disproportionately affected by HIV and have higher morbidity and mortality rates than the general population [5]. This calls for the development of screening tools that look out for key population individuals seeking hospital services and offer them HIV testing services.

## Study limitations

This study had some limitations. First, the use of cross-sectional and routine program data that is captured in paper-based registers would not allow us to examine cause and effect. Secondly, transcription errors may have occurred during the process of data abstraction from registers to the electronic system and could lead to study biases. Another limitation was on variables used in the analysis in that some key HIV-positive predictor variables, like lifestyle, and education level, among others, were not evaluated because the study was limited to variables available in the HTS laboratory register. Lastly, the study included a limited number of key populations thereby affecting the generalizability of the results to them.

## Conclusion

We found that client characteristics such as age, marital status, HIV test entry point, first-time test, repeat test after 12 months, and TB status are potentially predictive of the outcome of HIV case finding in PITC settings in central Kenya. The factors highlighted as determinants of a positive HIV test in this study can be used to develop a screening tool to target high-risk clients for HTS in similar settings. To the best of our knowledge, no similar study using a large dataset from the three central Kenya counties has been done. The researchers intend to build a prognostic risk model based on the multivariate model that will be trained, tested, calibrated, and validated to have a predictive capability to classify HTS clients as either low, medium, or high risk in low HIV prevalence settings.

## Data Availability

The dataset used is a deidentified dataset with individual-level routine HIV testing data and is not currently publically available as it is the property of the Ministry of Health and the Government of Kenya. The dataset can be obtained from the corresponding author based on a reasonable request.

## Abbreviations

aPNS: Assisted partner notification services
ART: Antiretroviral therapy
CDC: Centers for Disease Control and Prevention
CHS: Centre for Health Solutions - Kenya
CI: Confidence Interval
FSW: Female sex workers
GBV: Gender based violence
IMCI: Integrated management of childhood illnesses
IPD: Inpatient department
MOH: Ministry of Health
KENPHIA: The Kenya Population-based HIV Impact Assessment
MSM: Men who have sex with men
NASCOP: National AIDS and STI Control Program
OPD: Outpatient department
OR: Odds Ratio
PEPFAR: President’s Emergency Plan for AIDS Relief
PLHIV: People living with HIV
PMTCT: Prevention of mother-to-child transmission of HIV
PITC: Provider initiated testing and counselling
PrEP: Pre-exposure prophylaxis
PWID: People who inject drugs
SNS: Social network strategies
SSA: Sub-Saharan Africa
STI: Sexually transmitted infections
SQL: Structured Query Language
TB: Tuberculosis
UNAIDS: The Joint United Nations Programme on HIV/AIDS
WHO: World Health Organization
